# Social Media Multidimensional Analysis for Intelligent Health Surveillance

**DOI:** 10.3390/ijerph17072289

**Published:** 2020-03-28

**Authors:** María José Aramburu, Rafael Berlanga, Indira Lanza

**Affiliations:** 1 Departamento de Ciencia e Ingeniería de los Computadores, Universitat Jaume I, E-12071 Castellón de la Plana, Spain; 2 Departamento de Lenguajes y Sistemas Informáticos, E-12071 Castellón de la Plana, Spain; berlanga@uji.es (R.B.); lanza@uji.es (I.L.)

**Keywords:** health surveillance, social network analysis, multidimensional analysis, text mining

## Abstract

*Background*: Recent work in social network analysis has shown the usefulness of analysing and predicting outcomes from user-generated data in the context of Public Health Surveillance (PHS). Most of the proposals have focused on dealing with static datasets gathered from social networks, which are processed and mined off-line. However, little work has been done on providing a general framework to analyse the highly dynamic data of social networks from a multidimensional perspective. In this paper, we claim that such a framework is crucial for including social data in PHS systems. *Methods:* We propose a dynamic multidimensional approach to deal with social data streams. In this approach, dynamic dimensions are continuously updated by applying unsupervised text mining methods. More specifically, we analyse the semantics and temporal patterns in posts for identifying relevant events, topics and users. We also define quality metrics to detect relevant user profiles. In this way, the incoming data can be further filtered to cope with the goals of PHS systems. *Results:* We have evaluated our approach over a long-term stream of Twitter. We show how the proposed quality metrics allow us to filter out the users that are out-of-domain as well as those with low quality in their messages. We also explain how specific user profiles can be identified through their descriptions. Finally, we illustrate how the proposed multidimensional model can be used to identify main events and topics, as well as to analyse their audience and impact. *Conclusions:* The results show that the proposed dynamic multidimensional model is able to identify relevant events and topics and analyse them from different perspectives, which is especially useful for PHS systems.

## 1. Introduction

Public Health Surveillance (PHS) is defined as the ongoing systematic gathering, analysis, and interpretation of data, closely integrated with the dissemination of these data to the public health practitioners, clinicians, and policy makers responsible for preventing and controlling disease and injury [1]. In the last decade, many experiments have demonstrated that social media data can help public health officials to detect potential outbreaks, forecast disease trends, monitor emergency situations and gauge disease awareness and reactions to official health communications [2,3].

Traditionally PHS relied on surveys and primary data collected from healthcare providers and pharmacists on a weekly or monthly basis. While social media platforms cannot replace formal data sources for disease surveillance, they can provide complementary information with some advantages. Social media is a source of health and lifestyle information that covers all the society statements and is easy, rapid and cheap to obtain. However, for PHS, the filtering, integration and analysis of large amounts of data is as important as data gathering. The processing of social media data into useful information for public health action is a complex task and a main research topic [4]. In general, current approaches address specific problems, and propose powerful ad-hoc solutions for analysing information stored in isolated and static repositories of social data [3]. Consequently, their algorithms and results cannot be applied to data from different contexts. However, PHS agencies require the development of general tools with advanced mechanisms for extracting, integrating and analysing social media streams with a wide range of different purposes [2].

The main objective of our research is to provide PHS agencies with a new tool to analyse the evolution of posts from several positions of interest. Its main advantage is to make it easier to learn who is posting, about what and with what purpose, or any combination of these. This tool considers social media sites as continuous sources of relevant posts that are processed in real time to produce useful data. Then, users can execute operations to analyse the incoming data in order to monitor trends, and quickly identify events and alarms. Online analysis tasks consist of summarising the data at different levels of detail and from complementary points of view, depending on the purpose and the scope of the research. Furthermore, when needed, the pre-processed input data can be integrated with other data sources or stored in sandboxes dedicated to statistical analysis and historical studies. With this tool, PHS services can turn large amounts of posts into meaningful information, and easily and understand what is happening in social media in a timely manner.

## 2. Previous Work 

By analysing the latest advances of the Centers for Disease Control and Prevention, the report presented in [1] helps to understand the current state of development of PHS systems. In general, current systems present enhancements for data gathering (mainly, the integration of different data sources), data analysis and visualisation (mainly, online available data repositories), and dissemination (mainly, specialized, periodically published reports). However, these systems rely on disease-specific approaches that inhibit efficiency and interoperability and use out-of-date technologies that no longer meet user needs for data management, analysis, visualization, and dissemination. A main recommendation of [1] is that advances in information technology, data science, analytic methods, and information sharing provide an opportunity to enhance surveillance. Social networks as a new source of data for PHS were not mentioned in this review, showing that current systems are in their early research stage.

### 2.1. Using Social Networks for PHS

In the literature, many works demonstrate the usefulness of social media data for PHS. A research review presented in [5] already confirmed that social networks can be used as platforms to track the spread of infectious diseases, as well as warning detection systems with faster response than official data, which can take one or two weeks to collect and process. The authors conclude that PHS based on social media will never replace traditional surveillance, but social networks provide complementary data to be integrated with traditional sources. In their review, apart from the large amount of noisy data, the main open issues are the lack of coverage and the bias in the collected social media data. As solution, they propose new filtering methods for data quality together with advanced frameworks to integrate data from different social media platforms. 

In a complete and recent review [3], Twitter data has been found to be useful for several different public health applications, including monitoring diseases, public reactions, outbreak/emergency situations, prediction, lifestyle, geolocation, and other general applications. Authors highlight that most work done to date is not applicable to many use cases. They claim for a system that can be applied across researchers to categorize tweets in real-time is very important in order to better track information and facilitate rapid decision making. As in [5], the lack of coverage and the bias in the collected social data are the main drawbacks. However, in contrast to [5], they also consider that users are more important than tweets in public health research and recommend future studies to switch their unit of analysis from tweets to individual users. 

As reviewed in [3], many previous approaches successfully apply Machine Learning (ML) tools in order to manage large amounts of data at tweet level. Most of them use supervised ML methods that need manually annotated data collections. These systems classify posts in several ways, as for example: (i) cleaning noisy data with adverse drug reactions (ADR) [6] or with medical conditions [7]; (ii) observing the frequency and distribution of textual ADR mentions [8], and identifying ADR semantically annotated data [9]; (iii) grouping tweets by topic [10]; and (iv) assigning a polarity to both tobacco-related tweets [11] and posts about personal sentiments [12] or influenza [13].

In the literature, there are also some papers that apply Text Mining for identifying tweets’ contents, such as topic modelling and Latent Dirichlet Allocation (LDA) [3]. For example, in [14], a new associative topic model that identifies relevant tweets by using a combination of keywords and associated topics, obtained good results for monitoring diseases. In [15], unsupervised LDA-based clustering using topic modelling performed well for detecting relevant Twitter data. Compared to a trained classifier, the clustering method was found to offer less control over the topics, but the classifier was costlier because it required many manual annotations. Another LDA method of analysis presented in [16] was applied to large amounts of tweets in order to find topics related to public health. Their research results showed that only very common topics were detected.

Following the recommendations in [5], it is important to note that many efforts have focused on processing the posts independently and ignore the fact that the value of a post is inherently related to the credibility of its poster. At this level of analysis, the paper in [17] proposes a ML framework to classify social media users, which relies on data from user profile accounts, user tweeting behaviour, textual contents, and social network parameters. The framework was successful in identifying political affiliations, but failed in identifying user races. Similarly, in [18], an approach for enhancing PHS with quality social media data coming from trustworthy users is presented. This work proposes six trust filters to rank social media users with respect to a given criteria. Preliminary results show that the best filters are based on the number of related posts a user sends. The profiling of Twitter’s users to enhance tweet classification and relevance was already proposed as an open issue in [19].

### 2.2. Multidimensional Analysis of Tweets

In this section, we review some few works with similar purposes to ours, that is, works that process tweets in order to support multidimensional analysis.

In [20], authors propose an active surveillance methodology based on four dimensions: volume, location, time and public perception. They explore the public perception dimension by performing sentiment analysis and a clustering approach is used to exploit the spatio-temporal dimensions. Authors show that Twitter could be used to predict, spatially and temporally, dengue epidemics. They also propose a dengue surveillance approach that produces a weekly overview of what is happening in each city compared to the weeks before.

The M-Eco system, presented in [21], collects data from social media and TV/radio for public health monitoring purposes. It allows a user to search for disease names or symptoms and to assess the related signal information by means of a geographic map, a tag cloud or a timeline. Previously, texts are automatically annotated to identify diseases, person and location names. ML algorithms detect patterns in the data and, since the large number of generated signals can overwhelm a user, recommendation techniques are exploited to filter out those signals that are of potential interest for a user. Finally, the information is shown in charts and through personalized tag clouds to allow users to easily assess signals. Together with the frequent changes in posts terminology, again, the lack of social coverage and noisy data are identified as main limitations.

Considering the spatial dimension of tweets, in a recent work [22], Twitter data are used to extract spatio-temporal behavioural patterns to monitor flu outbreaks and their locations. In addition, they propose future extensions to study the epidemic spread of flu within different subpopulations by leveraging socio-economic and demographic data. Their results verify that flu-related traffic on social media is closely related to actual flu outbreaks. More specifically, they also find that clinical flu encounters lag behind online posts, and identify several public locations from which a majority of posts initiated. The main limitations of this study are very similar to previous works. The large amount of noisy data requires advanced classification techniques or machine learning approaches for deeper content analysis of social media posts. Furthermore, because not all users have their GPS enabled or declare their location in their social media profiles, spatial analysis of social media may be biased. The lack of GPS labels in posts is an open problem that some systems have already tried to solve by processing posts contents and metadata [7,10,23,24].

### 2.3. Modern Related Technologies

In our work, with the purpose of analysing social media contents from several positions of interest, we need to process the relevant posts in order to produce useful multidimensional attributes. Then, these parameters can be applied to summarise tweets and posters’ data at different levels of detail and to analyse social networks data from complementary points of view. As we have seen, none of the previous works have proposed the development of a system with this functionality. Such a solution requires the application of advanced information technologies. This section reviews the main technologies that our approach takes advantage of. More specifically, it reviews the following technologies: Business Intelligence, data quality, intelligent processing of data, and finally, streaming technologies.

#### 2.3.1. Business Intelligence

The main objective of Business Intelligence (BI) is extracting strategic knowledge from the information provided by different data sources to help companies during decision-making. The processing and analysis of massive data oriented to BI has evolved in recent years. Traditionally, the most commonly used approaches have combined data warehouse, online analytical processing (OLAP), and multidimensional design technologies [25]. OLAP tools were introduced to ease information analysis and navigation from large amounts of transactional data. These systems rely on multidimensional data models, which apply the fact/dimension dichotomy. Multidimensional data are represented as facts, whereas dimensions define hierarchies with different detail levels to aggregate data.

BI systems work on very specific scenarios, making use of static and well-structured data sources of corporate nature, and causing all the information to be fully materialized and periodically processed in batch mode for future analysis. More recently, new technologies for Exploratory OLAP have been introduced, aimed at exploiting semi-structured external data sources (e.g., XML, RDF) for the discovery and acquisition of relevant data that can be combined with corporate data [26].

Social networks have become a new source of valuable information for companies, helping them, among others, to know the opinions of their customers, to analyse market trends, and to discover new business opportunities [27]. The main purpose of Social Business Intelligence (SBI) is to help managers in making decisions by performing a multidimensional analysis of the relevant information disseminated on social networks. SBI and OLAP tools can enable the definition of hierarchies of analysis that organise and classify posts and users from complementary points of view and at different levels of detail [28,29]. Thus, from our point of view, by processing the posts relevant for PHS tasks, it is possible to build the multidimensional metadata structures applied by OLAP operators to aggregate social media data. At the end, BI systems can allow analysts to obtain many different visual representations that summarise the reality expressed in a large amount of posts.

#### 2.3.2. Data Quality

When dealing with social media data, the quality of data becomes a main issue [30]. Users and posts require measures to assess their usefulness for health surveillance. Measures such as the number of replies, likes, or retweets are indicators of the relevance of the posts. Combining them with parameters expressing the credibility of the sending user (e.g., number of followers or verified account) is also useful for cleaning up social media data. Posts without meaning or acceptance, as well as posts coming from users out of context or without credibility should be considered noisy data and be discarded by the system.

Furthermore, as pointed out by [3], retrieving relevant posts using a list of keywords might be problematic. The reason is that many tweets are relevant, but do not mention the predefined words, whereas many tweets including the keywords may be irrelevant for different reasons (misunderstandings, bots, spams, etc.). The main conclusion is that, after defining an initial list of keywords, the quality of the retrieved collection needs to be analysed with two main purposes, first, to find the best group of keywords, and second, to remove noisy posts [28,31,32].

In the literature, quality measures are defined at post and user level. At post level, there are many metrics covering the characteristics of the text (e.g., grammar, contents and semantics), together with the metrics specific to micro-blogs that reflect their social impact (e.g., number of retweets). On the other hand, at user level, there are activity metrics to assess the posters’ relevance (e.g., account age and number of posts) and popularity (e.g., number of followers, likes and mentions). 

The evaluation of the quality of contents published on micro-blogging platforms has focused mainly on post-retrieval operations. Searching for posts related to a topic [33,34]; filtering posts based on their credibility and quality [35,36]; detection of events and disasters [37,38,39]; analysis of feelings, political and consumer opinions [40,41,42]; and the detection of influencers [43,44], are some example applications. Other applications aimed at the detection of spammers, bots and advertising campaigns have proposed intelligent analysis techniques for social metrics [45,46,47]. 

#### 2.3.3. Intelligent Processing of Data

Entity resolution, topic classification and user profiling are intelligent processing tasks to extract meaningful data and semantically annotate posts. There are many types of meaningful data, such as the subjects and topics of posts, user affection or expertise, as well as post intent, and text polarity [29]. These automatic processes may apply different complex techniques [48]: crawler meta-data, information extraction, information retrieval, natural language processing, machine learning, and domain knowledge resources [4]. 

In recent years, we have witnessed a great interest in massively annotating the biomedical scientific literature [28]. Most of the current annotators rely on well-known lexical/ontological resources such as MeSH, Uniprot, and UMLS. These knowledge resources usually provide both the lexical variants for each inventoried concept and the concept taxonomies. Most semantic annotation systems are dictionary look-up approaches, that is, they rely on the lexicon provided by the ontology in order to map text spans to concept lexical variants. Although in a simpler way, the same techniques can be applied to find the topics associated to PHS-relevant posts.

In social media applications, opinion mining and sentiment analysis have been important research areas that combine techniques from Machine Learning (ML) and Natural Language Processing (NLP). One of the most relevant applications of sentiment analysis is aspect-based summarization [49]. Given a stream of opinion posts, aspect-based summarization is aimed at extracting the most relevant opinionated aspects along their sentiment orientation, usually represented as a score and a polarity. Aspect-based summarization has been usually divided into three main tasks, namely: sentiment classification, subjectivity classification and aspect identification. The first one is focused on detecting the sentiment orientation of a sentence, the second one consists of detecting if a sentence is subjective (i.e., if it contains a sentiment), and the latter one consists of detecting the most relevant aspects of an opinion stream. ML-supervised approaches have been widely adopted to solve these problems, because they can be easily modelled as traditional classification problems. Unfortunately, it is unfeasible to get training examples for all the items and potential aspects regarded in opinion streams. Thus, supervised approaches have been restricted to obtain sentiment lexicons and to detect sentence subjectivity with them [49]. Sentiment analysis in open scenarios should rely on unsupervised or semi-supervised methods [50]. Moreover, for social media data, sentiment analysis must be combined with social network parameters, which measure the diffusion and popularity of opinions spread across social networks [51].

#### 2.3.4. Streaming Technologies

The methods and architectures for BI are evolving. Traditional architectures consist of large data warehouses that integrate various data sources into a data repository under a multidimensional scheme. Data processing is executed in batch, which causes late alerts and delays in decision-making. A newer approach, in accordance with the current needs for Big Data processing, focuses more on the speed and immediacy of information, processing data in streaming and, when needed, building sandboxes where to execute batch analysis processes. In this way, only the data items needed for the knowledge models are stored, optimizing memory usage. 

Modern software architectures and programming technologies for real-time processing can be applied to process posts as soon as they occur. Some authors [29,52] propose an extended Lambda Architecture for Big Data processing [53] that includes semantic data processing and establishes mechanisms to semantically enrich raw data with metadata from various sources. Furthermore, it is necessary to adapt the hierarchies of analysis and the semantic processing of post to the dynamic behaviour of social media sites. This requires mechanisms to discover and add new aspects of analysis on the fly.

### 2.4. Conclusions

Summarizing this review, there are some unresolved problems that limit the utilization of social media data for PHS, namely: social coverage, bias in the available data, poor quality and noisy data, lack of information about users, language and multilingualism, etc. However, by applying computational intelligence and modern technologies, a large amount of useful information (e.g., topics, news, needs, events, sentiments) can be extracted from social media, to be processed and delivered for many applications. In [2], three major applications for social media in PHS are identified: epidemiologic monitoring, situational awareness during emergency response and communication surveillance. Therefore, as a general conclusion, we consider that it is time to provide health officers with intelligent tools prepared for the multidimensional analysis of social media data about a wide range of ailments, and with interactive functionalities for many surveillance tasks.

## 3. Methods

Although not everyone shares health conditions in social networks, they are so widespread that their study can serve PHS officers as a complement to traditional data sources [21,54]. The relevant aspects of social media posts for health are the following ones:

- *Sender users*. Active users in social media can be individuals (i.e., personal accounts that range from health professionals to lay users) as well as organizations (i.e., accounts belonging to associations, laboratories, official bodies, etc.). Lay users post mainly about their symptoms, diagnosis, announcements, opinions, and sentiments. However, collective users representing their organizations post journal news, recommendations, official announcements, events, promotions, etc. Organizations have a mission and a specialty. For example, the user named Cancer Discovery sends tweets with high-impact articles and news on major advances in cancer research, and the World Health Organization is committed to promoting good health habits. For organizations, the activity level in social media (i.e., number of posts and social media campaigns) and the credibility (i.e., number of followers and verified accounts) is usually much higher than for individuals. From a PHS perspective, individuals could be classified by degree of affectation (as patients, familiars, or interested) as well as by degree of knowledge (as health masters, specialists or professionals);
- *Posts contents*. The words in the posts can be applied to retrieve and filter them, but also to annotate them with the topics that describe their contents at different levels of detail. Topics can range from the most general subjects (i.e., medical condition, health campaign, financial service, etc.), to general topics (i.e., skin disease, WHO alert, AIDS treatment, etc.) up to very specific topics (i.e., nodular melanoma, sun protection recommendations, etc.). Real-world named entities, such as persons, locations, organizations, ailments and drugs, are also annotation elements that can be automatically identified. Post metadata elements like user locations, hashtags (# symbol) and screen names (@ symbol) should also be considered when annotating posts’ contents;
- *Posts polarity*. Many people choose social media to express positive and negative sentiments and opinions. Processing post contents into polarity values is useful to analyse social awareness and acceptance, or to detect alarms and conflicts in the population [19];
- *Posts temporality*. Users post their news as soon as they occur, so that social media is a source of timely information for PHS. The posts refer mainly to health issues that may occur uniformly and continuously, as well as seasonally or due to outbreaks. The temporal dimension of posts could allow researchers to filter them as well as to group them into different time units (i.e., daily, weekly, monthly, etc.). This would make easier to monitor the evolution of real facts, to detect reactions to announcements, to discover new information, and even to filter noisy data. For example, a peak in the distribution curve of posts about melanoma can serve to discover a huge amount of unusable posts reacting to the announcement of a famous person suffering from it.

In our approach, the intelligent processing of incoming posts automatically generates semantic annotations to represent different aspects. At the same time, this processing also allows for the filtering and classification of large amounts of social media data. Then, by combining one or more of these aspects, analysts can group the posts and summarise them in different ways.

For example, PHS officers may be interested in monitoring public reaction to seasonal campaigns reporting on how to prevent melanoma. After retrieving the relevant posts by means of keywords, the resulting collection could be large and difficult to manage. With our approach, the advanced processing of these posts annotates them as sent by organizations, general lay users or patients, containing opinions with positive or negative polarity, etc. Then, by adding conditions on these aspects and on the temporality of the posts, it would be possible to obtain meaningful indicators that measure social reactions from different points of view.

Other aspects of social media users related to epidemiological and demographic studies include personal attributes such as race, age, sex, or geographic location. Lay users do not specify these data in their profiles; consequently, there have been many efforts to extract them from posts’contents [10,17,55]. Currently we do not regard these aspects in our research tools.

### Multidimensional Analysis of Twitter Streams

**Figure 1:**
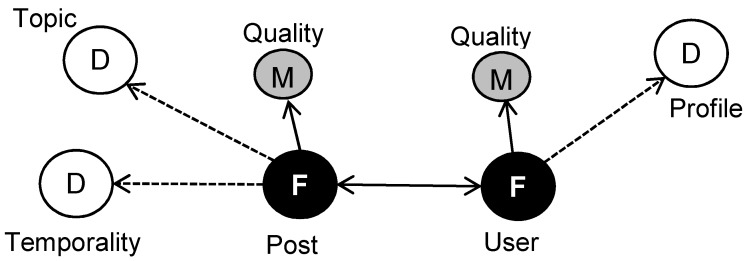
Summary of the multidimensional model for Twitter streams analysis. We follow the usual notation for multidimensional models: (D) for dimensions, (F) for facts and (M) for metrics. Dotted lines indicate that dimensions are dynamically created from the streamed data.

Our approach relies on multidimensional data models dynamically defined over Twitter streams [31]. More specifically, for PHS, we propose the star-based schema shown in Figure 1. The main purpose of this schema is to generate summarized topics and events from the stream so that they can be analysed as required by PHS tasks.

As can be observed, social media data elements are modelled with two different fact types. Post Facts consist of metrics extracted from post contents, manly topics, temporal attributes and other tweet quality parameters like number of replies or retweets. From a different perspective, User Facts include profile metrics extracted from descriptions and other user level quality parameters, like if the sending account is officially verified. Notice that some user metrics can be calculated by adding up the post metrics of all the tweets sent by the same user. The double-headed arrow connecting Post and User facts represents the binary correspondence that exits between the posts of a user and the poster of a tweet. This correspondence is needed to calculate some metrics, like the total number of replies of the relevant posts sent by a user.

In the proposed model, dimensions are dynamic in the sense that their values are derived from the incoming data. For example, the Topic Dimension accounts for the main subjects the tweets are discussing, which are statistically inferred after a certain number of tweets have been processed. More specifically, in this paper we propose a bigram-based temporal analysis to detect these topics in the stream of tweets. Furthermore, the temporal behaviour of the published information is quite relevant to characterizing posts. For example, a tweet can take part of a relevant news event, a health awareness campaign, or it can just be an isolated opinion. Thus, identifying the Temporality Dimension of posts allows us to properly group and analyse the information of the stream.

A relevant aspect included in our model is the quality of posts and users. Quality Dimension parameters can be derived from a combination of both, metrics provided by Twitter in the posts metadata (e.g., number of replies of tweet or the number of followers of a poster) and post contents metrics (obtained by processing the textual part of the posts). The resulting set of quality metrics indicates how reliable the information is with respect to the analysis at hand. For example, many users that publish a high quantity of posts can be spammers whose messages produce a great bias over some topics. Some messages can be just noise due to keyword ambiguity or more complex aspects like irony, sarcasm and metaphors. This last aspect is well known in the literature because some medical conditions like cancer and metastasis are often ironically used in other domains like politics. Quality metrics aim to assess how well the processed information fits with the analysis domain.

Finally, we include a Profile Dimension for users in order to identify their main intentions within the social network. Profiling users is very useful for social network analysis since it allows us to characterize both those who are posting information and those who are interacting with information. For PHS, we would like to dynamically distinguish between journalists, health-care professionals and services, as well as concerned people. Concerned people are individuals that have some true relationship with the health conditions being tracked (e.g., cancer survivors, relatives of patients). Profiling is performed over the brief text provided by the users in their Twitter accounts to introduce themselves. More specifically, we first identify and manually label a set of users by extracting meaningful frequent bigrams from their descriptions. Then, we train a neural-network classifier with the labelled users to predict the profiles of the incoming users.

In order to demonstrate the usefulness of the proposed streamed multidimensional analysis model, we designed a Twitter multi-keyword stream involving several topics from the PHS domain (see Table 1). The aim of the query is to reproduce a large and heterogeneous stream of tweets to monitor.

For analysing users, we define two quality metrics according to the information they publish, namely: *domain coherence* and *vocabulary diversity*. Both metrics rely on the language model derived from the tweets, that is, the distribution of their words. For domain coherence, we calculate the joint distribution between the user’s language model and a distribution of medical terms from UMLS Meta Thesaurus®. In our experiments, UMLS achieved a great coverage of medical terms, including the most common forms used by lay users to refer conditions and diseases. For vocabulary diversity, we propose the Yule’s I metric [56] applied to all terms (not only UMLS).

## 4. Results and Discussion

By using the Twitter API search method with the keywords shown in Table 1, we collected a large stream of tweets from June to December 2019. In this stream, a total number of 777,778 posts were tweets published by 395,804 users, where 77% of them posted just once in the stream. Concerning the overall quality of the stream, it is worth mentioning that the collected data are quite redundant as only 281,260 tweets consisted of unique texts, the rest being copies of other tweets. This fact shows how easily some streams could be biased towards the most repeated tweets. Finally, it is important to note that the retweets in the stream were treated separately, with the sole purpose of analysing user interaction.

### 4.1. Analysing Users

**Figure 2:**
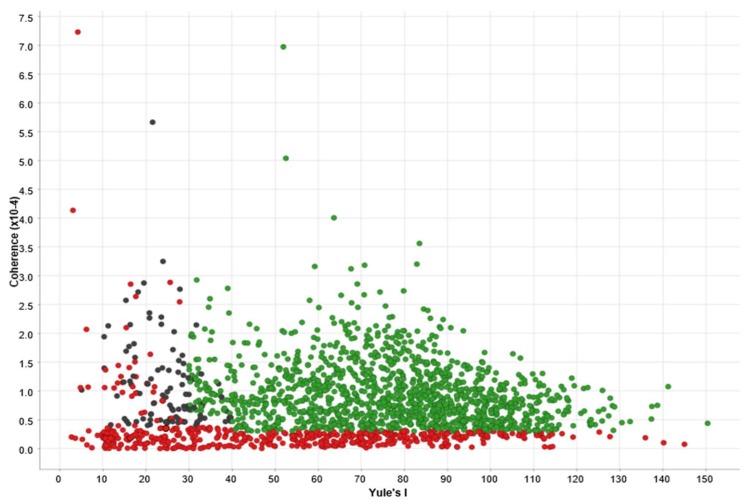
Quality metrics for the top-2000 users according to published tweets in the stream. Green points are good users, black points are users that need manual inspection, and red points are users that should be filtered out.

Figure 2 shows the user quality metrics for the top users according to the number of published tweets in the stream. Bots and spammers usually have a high number of tweets with a very small Yule’s I score (lower than 30). Users with very low domain coherence and a high Yule’s I score are usually prolific authors whose screen names contain some of the keywords of the stream, but their tweets are out of domain. By using these metrics, we can easily filter out non-relevant users (red dots in Figure 2). Notice that removing them implies rejecting around 15% of tweets from the original stream. Further details about the top users’ metrics are shown in Table 2.

### 4.2. Events and Topics

In the tweet stream, health-related events and topics are identified as bigrams grouped according to their temporal distributions of occurrence at day level. More specifically, we establish three thresholds, namely: a minimum kurtosis (10), a maximum peak of occurrences (20), and a maximum time span where the bigram occurs (10). As a result, around three thousand bigrams were selected. As these bigrams usually co-occur in many related tweets, we cluster bigrams by applying a graph modularity algorithm [57], resulting in 260 groups representing the full range of topics and events reported by the incoming tweets. It must be noticed that this process is fully unsupervised, and it aims at capturing the relevant events and topics of the stream. The detected events were manually inspected through web searches in order to confirm the results of the clustering method, and to demonstrate the usefulness of the quality metrics.

The impact of event-related tweets is around 7% of the total of tweets, whereas topics cover 51% of the incoming tweets. Additionally, 60% of event-related tweets have been also associated to some topic. Consequently, when analysing some topics, we must be aware of event-related tweets as they can have a high impact.

Table 3 and Table 4 show the top frequent events detected for posts written in English and Spanish, respectively. Events are usually related to some media news involving famous people from politics or sports. In Spanish, many events are related to children whose cases become viral. In these tables, we use the following quality metrics: *Y* (average Yule’s I), *%Ver.* (percentage of verified accounts) and *OnDom.* (average number of tweets per user in the stream). Furthermore, the last column shows the number of users tweeting and retweeting about these events.

The number of selected bigrams for topics is similar to that for events, around four thousand bigrams. These bigrams can be organized into 139 groups. Table 5 and Table 6 show the main topics according to the most frequent bigrams associated to different types of cancer for English and Spanish, respectively. It is important to notice that some few topics like “leukemia” and “melanoma” have been defined with unigrams instead of bigrams as their semantics are unambiguously associated to one single word. It must also be said that topics contain those events with the corresponding topics, so we must be aware of the impact of these events in the topics (e.g., “leukemia” topic). Notice that the users involved in topics are usually more active than those involved in events, mainly due to awareness campaigns associated with these topics.

Some interesting features we analyse are the use of first person and the percentage of replies associated with each topic (last columns of Table 5 and Table 6). It is worth mentioning that around 4% of tweets in the topic melanoma are indeed related to politics. This fact has been easily detected by exploring the most frequent subtopics (bigrams) associated with melanoma. In this case, we find out that 70% of the corresponding tweets are indeed replies, in contrast to the percent of replies of the whole melanoma topic (14%). On the other hand, the use of the first person indicates the ratio of people directly affected by the diseases.

**Figure 3:**
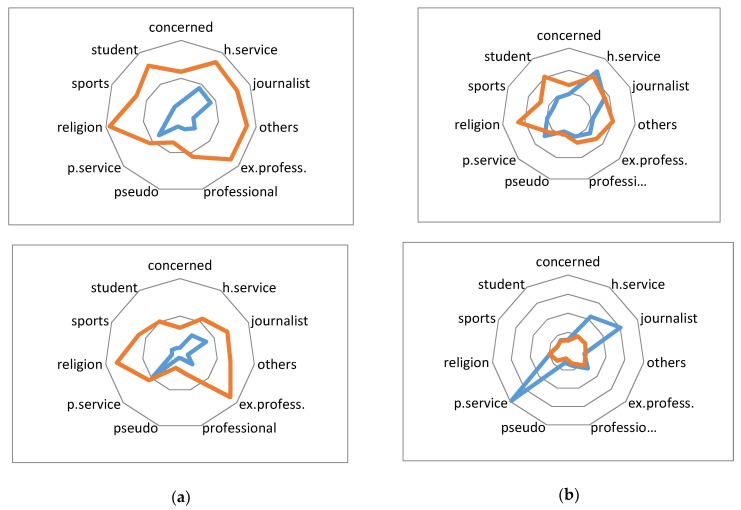
Comparing profiles from different topics and events (authors in blue and audience in red): (**a**) Top: Leukemia (topic), Bottom: Carlos Carrasco (event) (**b**) Top: Skin Cancer (topic), Bottom: Tapeworm (event).

Finally, Figure 3 serves to analyse the authors (in blue colour) and audience (in red colour) of some topics and events grouped by user profiles. As explained in Section 3, authors are identified as the posters of the relevant tweets, whereas the audience includes those users interested in retweeting the same tweets. Then, according to the language models of their descriptions, users can be classified into different profiles. Table 7 shows the language models obtained for these profiles.

Notice the different nature and impact of the profiles associated to each kind of event and topic. At the left side of Figure 3, the posts about the “leukemia” topic and the event reporting the “Carlos Carrasco” case have a clear origin in journalists and public or health services. It also shows a uniformly distributed large audience, except for alternative therapy users (pseudo in Figure 3). At the other side of the figure, the “skin cancer” topic has more different types of authors, including the large number of tweets created by public and health services, probably as part of prevention campaigns. Finally, the rare case of the “tapeworm” has been reported by many authors (probably trying to clarify its meaning or to prevent its consequences) but retweeted by a small audience.

### 4.3. Controversial and Dicussion-Related Topics

Finally, to show the usefulness of the proposed analytical model, we have selected a group of events and topics whose parameters show that they are candidates for monitoring during PHS tasks. Table 8 shows some topics and events with a low user quality and relevant audience. Notice that, except for the first topic, none of them have verified accounts in their audience. Topics 1 and 2 take the part of campaigns. Topics 3 and 4 present very low Yule’s I scores and a high number of messages in the same stream. These are clearly spam topics, used as a claim with non-informative purposes. Finally, Topic 5 is an example of an unverified event about some miraculous cure.

Regarding the nature of the tweets associated to these events/topics, the topic “black skin” associated to “skin cancer” has 43% of replies, indicating that is a very active discussion topic. The topic “force chemo” has only 3% of replies with few isolated opinions, which indicates a weak reaction against a government decision. The rest of topics/events in Table 8 do not contain any replies, despite there being many users writing about them.

### 4.4. Discussion

Nowadays, by applying modern Social Business Intelligence technologies, many organizations are developing solutions that integrate social media data into the multidimensional models of their decision-support systems. Here, one of the most important challenges is to adapt the analysis models to work with dynamic data streams and, in this way, to build systems that produce information in real time. The main contribution of the work presented in this paper is to apply this technology to the development of health surveillance systems. In this sense, the paper proposes a multidimensional model for Twitter streams analysis designed to exploit public health information in an intelligent way and demonstrates its usefulness for PHS tasks.

The proposed model annotates posts with attributes and metrics that describe their contents, users, quality and temporality. Then, these semantic annotations can be applied to filter and summarise the incoming data stream. The resulting system enables us to detect and track social media topics and events during PHS tasks. One strong point of the proposed method is the inclusion of quality metrics in the multidimensional model so that PHS analysis can be performed in a reliable way. Another strong point is the use of unsupervised statistical methods for identifying events, topics and profiling users. In this way, the multidimensional model can be adapted in real time to the data stream being monitored.

The use cases analysed in this paper demonstrate the advantages of the proposed tool for intelligent processing of social data and value creation in the context of PHS. With this conceptual framework, it is possible to derive a variety of dimensions to explain events and topics monitored by processes on demand. This is thanks to the linking and summarizing of the user-generated data (posts facts) to the users’ actions and profiles (user facts). The proposed methodology allows health officers to address the main applications of social media in PHS, like social monitoring, situational awareness and communication surveillance. 

The analysed use cases also show how to get insights into the most significant topics and events related to the tracked themes on Twitter, focusing on the nature and quality of the involved users. Analysing by author dimension and quality metrics allows us to elucidate the credibility of the source, for example to determine if the information comes from official sources, human users or bots. Grouping posts metrics by frequent relevant topics facilitates a quick inspection of possible disease outbreaks as well as related events. For example, in our experiments the statistics of tweets and replies written in the first person grouped by the topic “leukemia” allowed us to locate self-reported experiences in therapy. On the other hand, event detection offers the possibility of estimating the current incidence of a disease. The same methodology could be used to detect self-reported humanitarian needs during disasters. Knowing the type of user that produces the information and its audience, and measuring their reactions to certain messages, makes it possible to understand how they relate to each other and to monitor the degree of awareness and perception of PHS events.

## 5. Conclusions

In this paper we have presented a novel method for the multidimensional analysis of Twitter streams aimed at helping PHS systems. The proposed method can automatically detect events and topics from the stream according to their semantics and temporal features, and to properly summarize their contents, as well as the users involved in publishing and interacting with them. Our experiments on a real long-term stream about cancer show the usefulness of these multidimensional models for PHS tasks. The proposed method places special emphasis on the quality of the data and the audience involved in the detected events and topics. 

Future work will focus on defining new interesting key performance indicators (KPI) for PHS so that they can be estimated from the proposed multidimensional method in combination with other external data sources [42]. We also plan to improve the automatic classification of events and topics into simple taxonomies by using either external knowledge resources (e.g., UMLS Meta-thesaurus) and/or advanced text-mining techniques [30].

## Figures and Tables

**Table 1:** Keywords for the Twitter stream for Public Health Surveillance.

**Conditions:** cancer, tumor, carcinoma, leukemia, lymphoma, metastasis, sarcoma, skin cancer, melanoma **Treatments:** chemotherapy, homeopathy **Observations:** UV, Cholesterol, LDL, MRI

**Table 2:** Top-10 users’ quality metrics. In the screen names column, stream keywords are in bold face. Shadowed rows correspond to out-of-domain users.

Screen Name (User)	#Tweets	Yule’s I	Coherence (10^−4^)	Description
QunolOfficial	3306	42,3	0,88	Forum
**ldl**_bot	2556	94,3	0,21	Comics bot
**Lymphoma**Papers	2110	75,4	1,39	Academic bot
treda10	1740	70,6	1,15	Company CEO
AvosFromMexico	1623	49,9	0,33	Food sales
EurekaMag	1194	71,2	1,87	Magazine
medvizor	1053	56,6	0,65	Forum
beechnutwx	1010	11,2	0,31	Weather bot
SnoMiddleForkWX	963	29,8	0,32	Weather bot
Sara_**sarcoma**	947	113,7	0,02	Influencer

**Table 3:** Top frequent events (English).

Event (bigram)	#Tweets	Main Topic	User’s Quality (Y, %Ver., OnDom.)	#Users Tweet./Retw.
Alex Trebek	2659	Pancreas cancer	(70, 31%, 6)	2200/4535
Carlos Carrasco	659	Leukemia	(72, 28%, 4)	589/2473
Tapeworm woman	554	False tumor	(65, 39%, 6)	437/332
Ross Perot	357	Leukemia	(85, 23%, 4)	335/1093
Bank holidays (chemo canceled)	86	Chemotherapy	(68, 0%, 5)	84/27

**Table 4:** Top frequent events (Spanish).

Event (bigram)	#Tweets	Main Topic	User’s Quality (Y, %Ver., OnDom.)	#Users Tweet./Retw.
Tabare Vázquez	921	Lung Tumor	(57, 27%, 6)	691/1521
Carlos Carrasco	854	Leukemia	(55, 33%, 5)	262/622
*Francia homeopatía*	338	Homeopathy	(59, 11%, 6)	293/638
Luis Enrique	282	Cancer	(71, 13%, 3)	102/1331
*fármaco evita*	108	Cancer	(61, 7%, 7)	103/84

**Table 5:** Top frequent topics (English subset).

Topic	#Tweets	User’s Quality (Y, %Ver., OnDom.)	#Users Tweet./Retw.	%Tweets (Replies, 1st_Person)
leukemia	27.000	(76, 6%, 12)	3.200/17.300	(16%, 12%)
melanoma	26.791	(78, 8%, 15)	2.700/3.364	(14%, 7%)
skin cancer	19.000	(72, 7%, 5)	8.219/23.022	(23%, 12%)
brain tumor	18.900	(70, 5%, 5)	6.091/11.268	(37%, 28%)
lung cancer	1.500	(75, 4%, 45)	401/396	(18%, 8%)

**Table 6:** Top frequent topics (Spanish subset).

Topic	#Tweets	User’s Quality (Y, %Ver., OnDom.)	#Users Tweet./Retw.	%Tweets (Replies, 1st_Person)
*leucemia*	12.390	(57, 8%, 4)	1.388/11.108	(23%, 7%)
*tumor cerebral*	4.451	(67, 5%, 3)	1.264/9.951	(23%, 16%)
*melanoma*	3.726	(60, 15%, 9)	208/421	(8%, 3%)
*cáncer mama*	2.800	(58, 4%, 6)	221/188	(17%, 12%)
*cáncer páncreas*	1.100	(57, 3%, 4)	259/242	(2%, 3%)

**Table 7:** Language models for different profiles according to the users’ descriptions.

Profile	Definition	Top Words in Users’ Descriptions
professional	Specialists	radiosurgery, oncoplastic, oncologist, haemato-oncology, consultants
ex-professional	Retired people	retired, former, senate, viet, colonel
student	Students	student, thesis, undergraduate, engineering, studying
sports	Sportman	runner, marathon, athlete, skater, rider
religion	Religious terms	amin, allah, savior, hindu, jesus
p.services	Public services	traffic, 24h, breaking, weather, protection
h.services	Health-care services	urgencias, screenings, specialties, uninsured, visitors
concerned	Disease-concerned people	survivor, reminder_ribbon, survivorship, warrior
pseudo	Alternative therapies	reiki, meditation, yoga, ayurveda, remedial
journalist	Press and publications	medline-indexed, issn, journal, indexed, open-access
others	Other people	zombies, yin-yang, weekends, voracious, virgo (all with low probs)

**Table 8:** Controversial events/topics.

Topic/Event	User’s Quality (Y, %Ver., OnDom.)	#Users Twitting/Retw.	%Tweets (Replies, 1st_Person)
1. black skin	(85, 5%, 2)	180/5915	(43%, 22%)
2. force chemo	(97, 0%, 5)	66/15	(3%, 0%)
3. *evita metástasis*	(24, 0%, 282)	109/185	(0%, 0%)
4. *fármaco frenar*	(25, 0%, 255)	115/366	(0%, 0%)
5. *desaparece oración*	(56, 0%, 4)	83/3	(0%, 0%)
